# Development and multicenter validation of chest X-ray radiography interpretations based on natural language processing

**DOI:** 10.1038/s43856-021-00043-x

**Published:** 2021-10-28

**Authors:** Yaping Zhang, Mingqian Liu, Shundong Hu, Yao Shen, Jun Lan, Beibei Jiang, Geertruida H. de Bock, Rozemarijn Vliegenthart, Xu Chen, Xueqian Xie

**Affiliations:** 1 Radiology Department, Shanghai General Hospital, Shanghai Jiao Tong University School of Medicine, Haining Rd.100, Shanghai, 200080 China; 2 Radiology Department, Shanghai General Hospital of Nanjing Medical University, Haining Rd.100, Shanghai, 200080 China; 3 Winning Health Technology Ltd., Shouyang Rd., Lane 99, No. 9, Shanghai, 200072 China; 4 Radiology Department, Shanghai Sixth People Hospital, Shanghai Jiao Tong University School of Medicine, Yishan Rd. 600, Shanghai, 200233 China; 5 Department of Computer Science and Engineering, Shanghai Jiao Tong University, Dongchuan Rd. 800, Shanghai, 200240 China; 6 University of Groningen, University Medical Center Groningen, Department of Epidemiology, Hanzeplein 1, 9713 GZ Groningen, The Netherlands; 7 University of Groningen, University Medical Center Groningen, Department of Radiology, Hanzeplein 1, 9713 GZ Groningen, The Netherlands

**Keywords:** Computational biology and bioinformatics, Imaging

## Abstract

**Background:**

Artificial intelligence can assist in interpreting chest X-ray radiography (CXR) data, but large datasets require efficient image annotation. The purpose of this study is to extract CXR labels from diagnostic reports based on natural language processing, train convolutional neural networks (CNNs), and evaluate the classification performance of CNN using CXR data from multiple centers

**Methods:**

We collected the CXR images and corresponding radiology reports of 74,082 subjects as the training dataset. The linguistic entities and relationships from unstructured radiology reports were extracted by the bidirectional encoder representations from transformers (BERT) model, and a knowledge graph was constructed to represent the association between image labels of abnormal signs and the report text of CXR. Then, a 25-label classification system were built to train and test the CNN models with weakly supervised labeling.

**Results:**

In three external test cohorts of 5,996 symptomatic patients, 2,130 screening examinees, and 1,804 community clinic patients, the mean AUC of identifying 25 abnormal signs by CNN reaches 0.866 ± 0.110, 0.891 ± 0.147, and 0.796 ± 0.157, respectively. In symptomatic patients, CNN shows no significant difference with local radiologists in identifying 21 signs (p > 0.05), but is poorer for 4 signs (p < 0.05). In screening examinees, CNN shows no significant difference for 17 signs (p > 0.05), but is poorer at classifying nodules (p = 0.013). In community clinic patients, CNN shows no significant difference for 12 signs (p > 0.05), but performs better for 6 signs (p < 0.001).

**Conclusion:**

We construct and validate an effective CXR interpretation system based on natural language processing.

## Plain language summary

Chest X-rays are accompanied by a report from the radiologist, which contains valuable diagnostic information in text format. Extracting and interpreting information from these reports, such as keywords, is time-consuming, but artificial intelligence (AI) can help with this. Here, we use a type of AI known as natural language processing to extract information about abnormal signs seen on chest X-rays from the corresponding report. We develop and test natural language processing models using data from multiple hospitals and clinics, and show that our models achieve similar performance to interpretation from the radiologists themselves. Our findings suggest that AI might help radiologists to speed up interpretation of chest X-ray reports, which could be useful not only in patient triage and diagnosis but also cataloguing and searching of radiology datasets.

## Abstract (web summary)

Zhang et al. develop a natural language processing approach, based on the BERT model, to extract linguistic information from chest X-ray radiography reports. The authors establish a 25-label classification system for abnormal findings described in the reports and validate their model using data from multiple sites.

## Introduction

Chest X-ray radiography (CXR) is one of the most frequently used and easily accessed radiology examinations for screening and diagnosing pulmonary and cardiac diseases^1^. The interpretation of CXR mainly depends on the observation and experience of radiologists. However, the growing demand for CXR has brought a burden on medical staff which limits the clinical application of CXR, especially in community clinics or primary hospitals^2^.

Convolutional neural network (CNN), the representative algorithm of artificial intelligence (AI), enables the computational model composed of multiple processing layers to learn image features for image classification and has been applied in many medical fields^3–5^. With the advance of CNN in identifying diseases on CXR, such as tuberculosis^6^, pneumothorax^7^, lung nodule^8^, lung cancer^9^, and COVID-19^10^, radiologists may read CXR with the assistance of AI. Several studies have reported that CNN models showed radiologist-equivalent performance in identifying multiple disorders on CXR^11–13^. However, there is still a lack of large-scale clinical implementation of AI-assisted multidisease simultaneous reading on CXR. Studies have been conducted to validate the performance and generalizability of applying AI to read multiple diseases simultaneously. Rajpurkar et al. validated a CNN model based on 420 CXR images from the ChestX-ray8 dataset and found that it was comparable to radiologists in identifying 11 pathologies^14^. Recently, Wu et al. compared the reading results of an AI algorithm and five radiology residents on 1998 CXR images of inpatients randomly selected from the National Institutes of Health (NIH) dataset and concluded that the algorithm may reach the level of third-year radiology residents^15^. Most of the previous studies tested CNN models on datasets from academic hospitals, which hardly represented the distribution of abnormal signs in other populations, because the real-world distribution of thoracic diseases is complex. The disease distribution is diverse in patients from academic hospitals and community clinics and different in symptomatic patients and screening examinees. As far as we know, there is a lack of large-scale multicenter studies that comprehensively verified the performance of CNN in identifying multiple abnormal signs on CXR in various patient groups.

The traditional CNN medical image classification model usually adopts a supervised training method based on expert annotation. Although radiology reports of CXR images contain valuable diagnostic information, they are usually composed of unstructured natural texts, which cannot be processed directly by conventional CNN. Natural language processing (NLP) provides a method of extracting words from radiology reports, which is suitable for finding keywords describing medical images, so as to realize automatic annotation of a large number of CXR images^16, 17^. Recently, the bidirectional encoder representations from transformers (BERT) was developed for NLP^18^. The BERT model has greatly improved the capability and performance of NLP in semantic and context recognition and achieved higher ExactMatch and F1-scores than human beings in the SQuAD 1.1, the top-level text comprehension competition based on machine learning^19^.

**Fig. 1:**
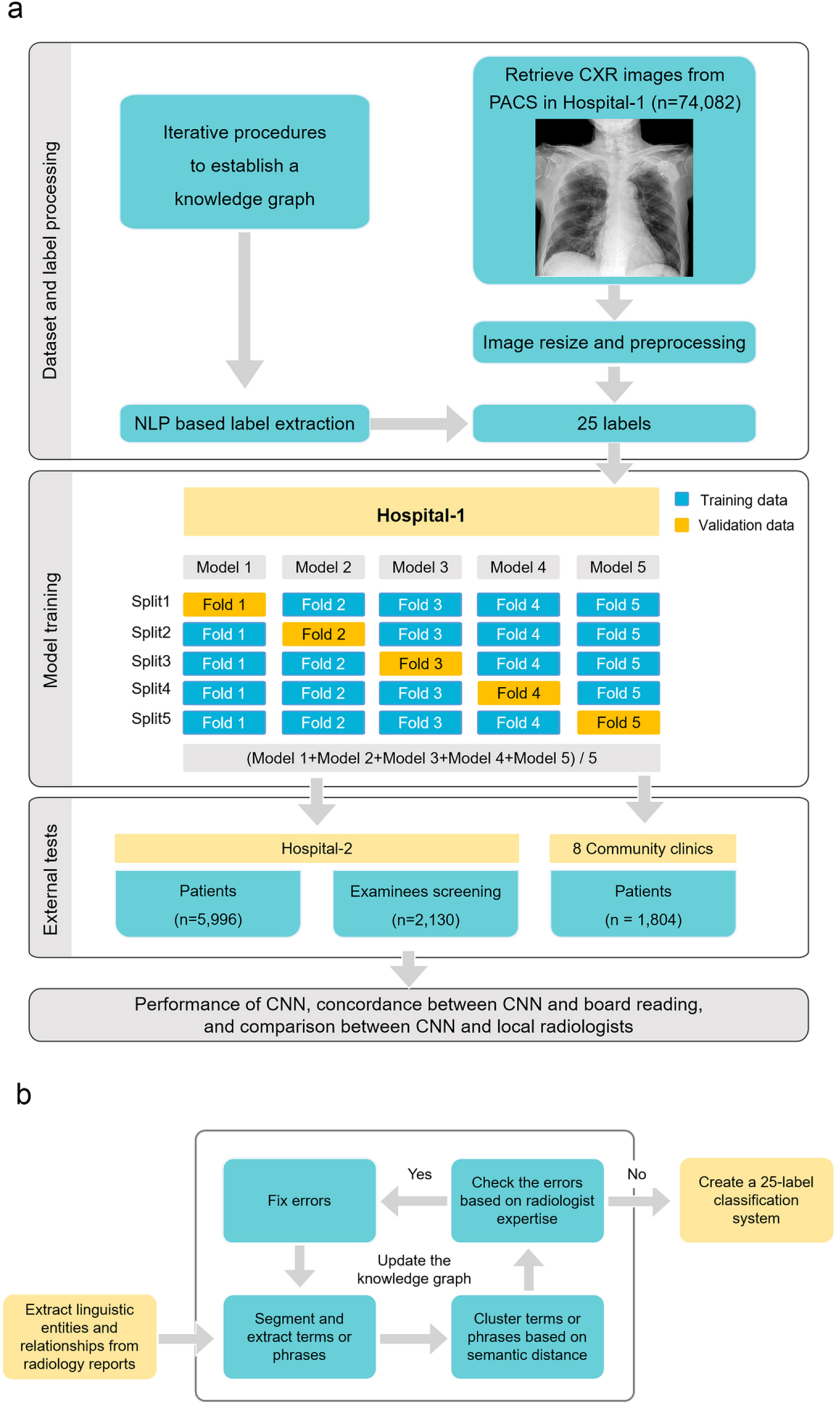
Diagram of research steps. **a** Workflow of dataset preparation, model training, and external testing. The training dataset consists of chest X-ray radiographs (CXR) and corresponding diagnostic reports. By using the bidirectional encoder representations from transformers (BERT) model to identify language entities from the reports, we conducted an iterative process to build a knowledge graph with the semantic relationship between language entities and finally established 25 labels representing 25 abnormal signs in CXR. After training the convolutional neural networks (CNNs) based on fivefold stratified cross-validation and weakly supervised labeling, we conducted external tests in another hospital and eight community clinics. The tests included the performance of CNN, the concordance between CNN and board reading, and the comparison between CNN and local radiologists. **b** Workflow of image labeling based on the bidirectional encoder representations from transformers (BERT) natural language processing model with an expert amendment. We used the BERT model to recognize linguistic entities, entity span, semantic type of entities, and semantic relationships between entities. In an iterative process to establishing the knowledge graph with the semantic relationship between language entities, two radiologists examined the established knowledge graph, amended the extracted linguistic entities, and clarified linguistic relationships based on their clinical experience. Finally, 25 labels representing 25 abnormal signs were established. CXR chest X-ray radiography, BERT the bidirectional encoder representations from transformers, CNN convolutional neural network, PACS picture archiving and communication system, NLP natural language processing.

In this study, we first collected a large number of CXR images and then trained CNN with weakly supervised labeling. This process used the BERT model to extract linguistic entities and relationships from unstructured radiology reports and constructed a knowledge graph to represent the association between image labels of abnormal signs and the report text of CXR. We evaluated the classification performance of CNN on three external test datasets from an academic hospital (symptomatic patients and asymptomatic screening examinees) and eight community clinics, accessed the classification consistency between CNN and radiologist consensus reading and compared the performance between CNN and local radiologists. The research steps are shown in Fig. 1. Finally, we established 25 labels representing 25 abnormal signs. In the three external test cohorts, the classification performance of CNN was slightly lower than that of local radiologists in an academic hospital but slightly higher than that in community clinics.

## Methods

### Population and study design

The cases as the training and tests data were from the Chest Radiography at Diverse Institutes (CRADI) dataset^20^. The population as the training dataset (*n* = 74,082) was retrospectively and consecutively collected from February 2014 to February 2018 in an academic hospital (Hospital-1, Shanghai Sixth People Hospital). The CXR image and corresponding diagnostic report of each subject were retrieved from the picture archiving and communication system (PACS) and radiology information system (RIS). The inclusion criteria were as follows: adults ≥18 years old with symptoms such as fever, cough, and chest pain, as indications for CXR. If a patient had multiple CXR examinations, only the earliest one was included. The exclusion criteria were: mobile CXR, insufficient image quality, and incomplete reports without the confirmation of a senior radiologist.

The cases in CRADI dataset as external test data came from another academic hospital (Hospital-2, Shanghai General Hospital) (*n* = 7797) and eight community clinics (*n* = 1804). The population was retrospectively and consecutively included from January 2019 to March 2019. The subjects in Hospital-2 were divided into symptomatic patients and screening examinee groups. The inclusion and exclusion criteria were similar to those of the training dataset, except that the screening examinees were asymptomatic.

After training the CNN models with weakly supervised labeling, we conducted a three-step external test on the CRADI dataset. First, we tested the diagnostic performance of AI-derived results using the radiologist consensus reading as a reference (see below). The tests were conducted in three external cohorts: symptomatic patients in Hospital-2, asymptomatic screening examinees in Hospital-2, and symptomatic patients in eight community clinics. Second, under the premise of using multi-label CNN classification for diagnosing abnormal signs on CXR, we evaluated the number of consistently classified labels between CNN and radiologist consensus reading in the three test cohorts. Third, the performance of CNN and local radiologists was compared. The Institutional Review Board of Shanghai General Hospital approved the use of the CRADI dataset in this retrospective study and waived the requirement of written informed consent.

### CXR equipment

Five different types of digital radiography systems were used to take posteroanterior CXR in Hospital-1 (Digital Diagnost, Philips; GC85A, Samsung; RADspeed, Shimadzu; CXDI, Canon; and XR220, Optima), four types in Hospital-2 (XR656, GE; Optima XR220, GE; Platinum 43, DMS; and CXDI, Canon), and five types in the eight community clinics (CXDI, Canon; DRX-Evolution, Carestream; uDR, United Imaging; Fluorospot Compact FD, Siemens; and Digital Diagnost, Philips). Supplementary Table 1 lists the DR equipments and acquisition parameters.

### Diagnostic reports

For each case of CXR in this study, a resident drafted a diagnostic report, and an experienced radiologist supervised to finalize it. In this way, 67 residents and 20 radiologists completed the reports in Hospital-1, 27 residents and 28 radiologists in Hospital-2, and 15 residents and 18 radiologists in the eight community clinics. Since the reports collected in this study were obtained in the actual clinical environment, all residents and radiologists had access to patient information, medical indications, and previous images.

### Construction of a knowledge graph with BERT

We used the BERT model^18^ to recognize linguistic entities, entity span, semantic type of entities, and semantic relationships between entities. BERT relies on a Transformer, an attention mechanism for learning the contextual relationships between words in a text. The BERT model is designed to pretrain the deep bidirectional representation from unstructured text through the joint adjustment of left and right contexts. Therefore, the pretrained BERT model can be finetuned by an additional output layer to create state-of-the-art models for various NLP tasks, such as learning the semantic information of the text and outputting vectors of semantic recognition which can be used for classification.

In this study, linguistic entities refer to words or phrases that describe anatomical region (such as lung, aorta, and hilum), the location of lesions on posteroanterior CXR (such as left, bilateral, and upper), image feature name (such as nodule and consolidation), and image feature adjective (such as blur and large). Relationship refers to the semantic and logic connections between linguistic entities, such as left-large-consolidation and bilateral-sharp-costophrenic angle. Two radiologists with 21 and 31 years of experience examined and amended the extracted linguistic entities according to the Fleischner Society’s glossary^21^ and a traditional radiology textbook^22^, and examined the linguistic relationships based on their experience. According to the anatomical region, the linguistic entities describing abnormal signs on CXR were divided into four categories: pleura, lung parenchyma, mediastinum, and thoracic wall. The representative linguistic entities and relationships from unstructured radiology reports are shown in Supplementary Fig. 1.

A knowledge graph^23^, also known as a semantic network, represents a network of real-world entities and illustrates the relationship between them. In building a knowledge graph, the pipeline method was used to extract linguistic entities and then analyze the relationship among candidate entity pairs. We defined and restricted the potential relationships through rules. For example, it is reasonable to have a direct semantic relationship between the anatomical region and image feature name, but the relationship between the anatomical region and image feature adjective is meaningless. The edges between two linguistic entities are also weighted to indicate whether the relationship is positive or negative. The extracted terms or phrases were classified as negative (e.g., “no abnormal sign”), positive (e.g., “consolidation in the inferior lobe of the right lung”), or positive with uncertainty (e.g., “possible” or “not excluded”). The tuples in the constructed knowledge graph imply rich semantic relations between different pairs of entities. For example, one tuple may show whether there is consolidation in the inferior lobe of the right lung or not.

In this study, the natural language data from different doctors were used to build image labels. When describing chest disorders, their language in free-text radiology reports has a hierarchical relationship. In order to understand the hierarchical relationship, we built the “anatomical position”—“disorder” relationship to help entity recognition and relation extraction. For example, for the two connections of “pleural thickening” and “pleural abnormality”, thickening is a subclass of abnormality. Building a knowledge graph helps us to understand the paths between different entities, form labels at different levels, and merge a large number of synonyms in the free-text reports. In the meanwhile, we can count the number and frequency of different entities, so as to reclassify the entities with a lower frequency to the upper level. For example, there were descriptions of “aortic arch calcification” and “aortic knob calcification” in the training dataset, both of which were less than their upper-level label “aortic arteriosclerosis”. Therefore, we can combine “aortic arch calcification” and “aortic knob calcification” into the label of “aortic arteriosclerosis” to train the models.

Finally, with the help of the constructed knowledge graph, we can configure complex query items to search target information. Keywords, terms, or other hand-designed conditions make it more accurate to determine labels of CXR images from the unstructured reports.

### Image labeling in the training dataset

CXR images in the training dataset were annotated according to the linguistic clusters extracted from radiology reports by the BERT model. Initially, from the perspective of the anatomical region, linguistic clusters consisted of four categories, including pleura, pulmonary parenchyma, mediastinum, and chest wall.

First, the BERT model mined all radiology reports in the training dataset, to extract all the terms with close entity span to construct linguistic clusters. The image descriptions and conclusions in radiology reports were combined into one source and then split into multiple sentences. The BERT model automatically segmented and extracted terms or phrases from these sentences, and clustered them according to semantic distance^24^. This process provided more than 40 linguistic clusters of synonyms or parasynonyms about abnormal signs on CXR, to identify the meaningful and frequent terms describing the abnormal signs.

**Fig. 2:**
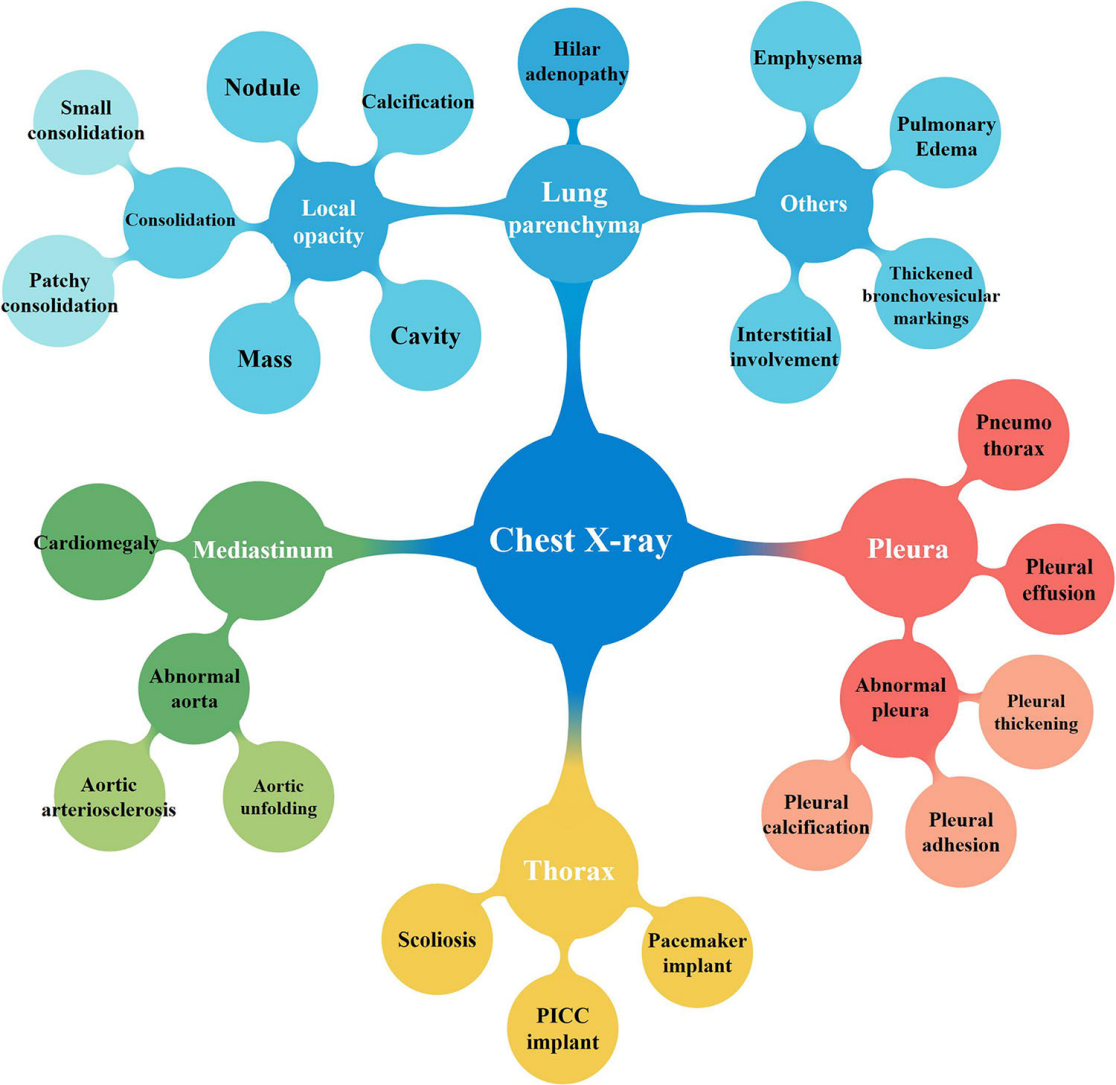
Diagram of 25 labels of abnormal signs extracted from the radiology reports by the bidirectional encoder representations from transformers (BERT) model. According to the anatomical region, the linguistic entities describing abnormal signs on chest X-ray radiographs were divided into four categories: pleura, lung parenchyma, mediastinum, and thoracic wall. The words in white color refer to anatomical regions or general categories. The words in black color represent labels of abnormal signs.

Next, the two abovementioned experienced radiologists and one NLP engineer reviewed the linguistic clusters to determine whether the terms in them correctly described the image findings on CXR. They decided by consensus whether a term or phrase belongs to a linguistic cluster according to its clinical meaning and dependence. They also iteratively ruled out wrong terms and fixed conflicting terms in this cluster, and merged clusters with similar clinical meaning. In this way, a linguistic cluster and its affiliated terms were updated. This process was iterated several times to optimize the knowledge graph until all the extracted terms or phrases were correctly categorized and associated. In the process of iterative error correction, if a linguistic cluster came from more than 50 subjects in the training dataset (except for cavity, which is rare but clinically important), then the cluster and its affiliated terms were regarded as a category of abnormal sign in the knowledge graph, and then used as a label to train CNN model, because the number of positive cases for training the AI model should not be too small. Finally, a knowledge graph of 25 abnormal signs was established (Fig. 2), which contained synonyms or parasynonyms, or phrases that may appear in natural reports. Then, a 25-label classification system were built to train and test the CNN model.

### Image labeling in the test datasets

Because the original natural language reports in the test cohorts cannot be directly used as a reference to compare with the results of CNN, we used the BERT model to extract the description of abnormal signs from the original reports. On the basis of the knowledge graph derived from the training dataset, the image labels of abnormal signs in the three test cohorts were extracted from the original reports. One radiologist with 10 years of thoracic imaging experience recorded and checked the BERT-extracted labels to ensure that they were consistent with the description of the radiology reports.

### Radiologist consensus reading

Because the original CXR reading was performed by medical staff with the various extent of expertise in different institutes, we applied an expert consensus reading to reclassified CXR images in the test datasets, in order to allow a high-standard and homogeneous reference standard. Two radiologists with 21 and 31 years of experience independently accessed the CXR images and BERT-extracted labels in the above step. They made necessary corrections to the label according to their clinical experience and resolved the inconsistency by consensus.

### Visualization of data distribution and CNN activation

We used the t-SNE algorithm to visualize the distribution of labels in the training and test datasets^25^. It successfully reveals the hidden structures, natural clusters, and smooth nonlinear variations along the dimensions in data. In the pooling layer of CNN architecture, all parameters are extracted and converted into a joint probability to minimize the Kullback–Leibler divergence between the original embedding and the low-dimensional embedding. The traditional t-SNE algorithm can only visualize one target^26^. However, a subject may not have or have one or multiple abnormal signs on CXR as the target of t-SNE. To visualize the natural clustering of abnormal signs, the signs of each subject were converted into a color index as the target of t-SNE, as follows: CI = *Y*_th_ × 8 + *Y*_me_ × 4 + *Y*_lp_ × 2 + *Y*_pl_ × 1, where CI is the color index, and *Y*_th_, *Y*_me_, *Y*_lp_, and *Y*_pl_ represent the one-hot representation of thoracic wall, mediastinum, lung parenchyma, and pleura, respectively. This one-hot representation can be taken as binary code, which can give each subject a color index. Thus, the normal subjects were coded as 0; the subjects with one abnormal sign in the thoracic wall, mediastinum, lung parenchyma, and pleura were coded as 8, 4, 2, and 1, respectively. A subject with multiple abnormal signs was coded as the sum of four independent codes. For example, a subject with abnormal mediastinal and lung parenchymal signs was coded as 6, with an abnormal thoracic wall, mediastinal, and lung parenchymal signs were coded as 14.

A class activation map (CAM)^27^ was generated to indicate the image regions that play a decisive role in CNN classification results. The active region for CNN classification was calculated by summing the weights associated with the feature maps in the final convolutional layer of CNN.

### Training algorithm and environment

The training model of this study was based on the inception-v4 CNN architecture^28^, which was pretrained on the ImageNet dataset. In the last fully connected layer of the inception-v4 architecture, the original 1000 classes were replaced by 25 classes, representing 25 abnormal signs on CXR. Because of the uneven distribution of positive and negative subjects under some labels in the training dataset, a customized weighted binary cross-entropy loss function was used to place greater weight on the infrequent input data.

We performed fivefold stratified cross-validation. Stratified cross-validation splits the data into multiple folds to ensure that each fold has the same observation ratio^29^. This method provides more information than one training-validation fitting model. In the five deep learning procedures, the entire training dataset was divided into 80% for training and 20% for internal validation. Then, in each cross-validation, 25 two-way CNN classification models were established to identify the 25 labels. Each model was trained for 24 epochs. The mean prediction probability of the five inception-v4 models was taken as the final prediction value. This mean value was then used to determine the threshold between positive and negative results by obtaining the maximum F1-score for each label. This threshold was then used to calculate sensitivity and specificity.

The code of study is available at https://zenodo.org/record/5335914^30^. We used an open-source tool for deep learning (PyTorch, http://pytorch.org/), and also used a computer vision library (opencv, http://opencv.org/), and a data analysis library (scikit-learn, http://scikit-learn.org/). The program runs on a Linux platform (Ubuntu 16.04, Canonical Ltd.) with four graphics processing units (GTX 1080Ti, Nvidia) in parallel with a total of 44 GB graphical random-access memory.

### Statistical analysis

The data following normal distribution are expressed as mean ± standard deviation. We employed five performance metrics to evaluate the results of the proposed CNN, namely area under the curve (AUC), accuracy, sensitivity, specificity, and F1-score. The 95% confidence intervals of these metrics were calculated by bootstrapping with 100 iterations to estimate the uncertainty of results^31^. In this way, the original data was resampled 100 times. Each time, 95% of the data were randomly selected and used to calculate the statistics of interest. The comparison between AUCs was conducted using DeLong’s test^32^. A two-sided *P* < 0.05 was considered statistically significant. A statistical software package (MedCalc v18, MedCalc Software) was used for statistical analysis.

## Results

### Study population and datasets

The data collection included two steps. First, the CXR images and radiology reports of 74,082 subjects (mean age 50.0 ± 17.1 years) from an academic hospital (Hospital-1) were retrospectively collected as a training dataset. Using the BERT model to extract the linguistic entities and relationships from the unstructured radiology reports, a knowledge graph was constructed to represent the relationship between CXR labels and report content, which laid the foundation for training CNN with weakly supervised labeling.

Second, to determine the classification performance of CNN for multiple abnormal signs on CXR, and compare with local radiologists in a multicenter clinical setting, we used the test cohorts of the CRADI dataset from another academic hospital (Hospital-2, Shanghai General Hospital) and eight community clinics, including 5996 symptomatic patients (aged 52.6 ± 16.7 years) and 2130 asymptomatic screening examinees (34.5 ± 13.6) from Hospital-2, and 1804 symptomatic patients (69.1 ± 14.0) from eight community clinics. Due to the low incidence of abnormal signs on CXR in real-world practice, a large number of subjects were included to test the performance of the CNN model. Using the BERT model for NLP, we extracted information from the radiology reports in the test cohorts of the CRADI dataset to represent the results of local radiologists. Because most CXR cases lack the gold standard of pathology, in order to establish a solid and unified reference standard to determine the performance of the CNN model, we conducted an expert consensus reading on the whole test cohorts. Table 1 shows the population characteristics of the training and test datasets.

**Table 1:** Study population characteristics of the chest radiograph at diverse institutes (CRADI) dataset.

	Training cohort		Test cohorts	
	Hospital-1	Hospital-2 (symptomatic patients)	Hospital-2 (screening examinees)	Eight community clinics (symptomatic patients)
Total number	74,082	5996	2130	1804
Male	34,828 (47.0%)	2964 (49.4%)	817 (38.3%)	673 (37.3%)
Female	39,254 (53.0%)	3032 (50.6%)	1313 (61.7%)	1131 (62.7%)
Mean age, years	50.0 ± 17.1	52.6 ± 16.7	34.5 ± 13.6	69.1 ± 14.0
Age range, years	18 to 102	18 to 102	18 to 92	21 to 101
Positive case	40,743 (55.0%)	2686 (44.8%)	206 (9.7%)	802 (44.5%)

### Establishment of a knowledge graph and CXR labels

After extracting linguistic entities and relationships from the radiology reports using the BERT model (Supplementary Fig. 1), we constructed a knowledge graph containing 25 abnormal signs found in the radiology reports. Each abnormal sign included synonyms, parasynonyms, and descriptive phrases that may appear in natural language reports. The linguistic entities contained in each abnormal sign were summarized as a label for training CNN; thus a total of 25 labels were defined for 25 abnormal signs (Fig. 2). The abnormal signs were mainly located in the lung parenchyma, mediastinum, pleura, and chest wall. The 12 labels of lung parenchyma included consolidation, small consolidation, patchy consolidation, nodule, calcification, mass, interstitial disease, cavity, hilar adenopathy, emphysema, pulmonary edema, and thickened bronchovascular markings. The four labels of mediastinum included cardiomegaly, abnormal aorta, aortic unfolding, and aortic arteriosclerosis. The six pleural labels consisted of pneumothorax, pleural effusion, abnormal pleura, pleural thickening, pleural adhesion, and pleural calcification, and the three thoracic wall labels comprised scoliosis, peripherally inserted central catheter (PICC) implant, and pacemaker implant.

T-distributed stochastic neighbor embedding (t-SNE) algorithm reduced the dimension of abnormal signs of four anatomical locations into a two-dimensional plane. In the training and three test cohorts (patients and screening examinees in Hospital-2 and patients from eight community clinics), t-SNE visualizes the distribution of disease labels extracted through the BERT model and shows the aggregation of abnormal cases (Supplementary Fig. 2 and Data 1).

### Training dataset

In the training cohort (*n* = 74,082), 33,339 (45.0%) cases were normal, 10,706 (14.5%), 5789 (7.8%), 5977 (8.1%), 4031 (5.4%), 4954 (6.7%), and 9286 (12.5%) cases had one, two, three, four, five, and more than five abnormal signs, respectively. The most common abnormal signs were thickened bronchovascular markings (*n* = 37,954), abnormal pleura (*n* = 13,085), pleural thickening (*n* = 12,789), nodule (*n* = 12,192), and consolidation (*n* = 9701).

The CNN classified 25 abnormal signs, and the AUCs of these abnormal signs ranged from 0.880 (95% CI: 0.878–0.883) to 1.000 (Supplementary Data 2). The mean AUC, accuracy, sensitivity, specificity, and F1-score of 25 signs were 0.958 ± 0.027, 0.943 ± 0.063, 0.607 ± 0.252, 0.958 ± 0.078, and 0.703 ± 0.192, respectively. In particular, the AUC of common signs such as consolidation, nodule, mass, pneumothorax, and pleural effusion were 0.940 (0.939–0.943), 0.880 (0.878–0.883), 0.985 (0.976–0.993), 0.971 (0.967–0.975), and 0.986 (0.985–0.987), respectively. The corresponding accuracies were 0.912 (0.910–0.914), 0.870 (0.868–0.872), 0.998 (0.997–0.999), 0.972 (0.971–0.973), and 0.986 (0.985–0.987), respectively.

### Symptomatic patients from an academic hospital

**Fig. 3:**
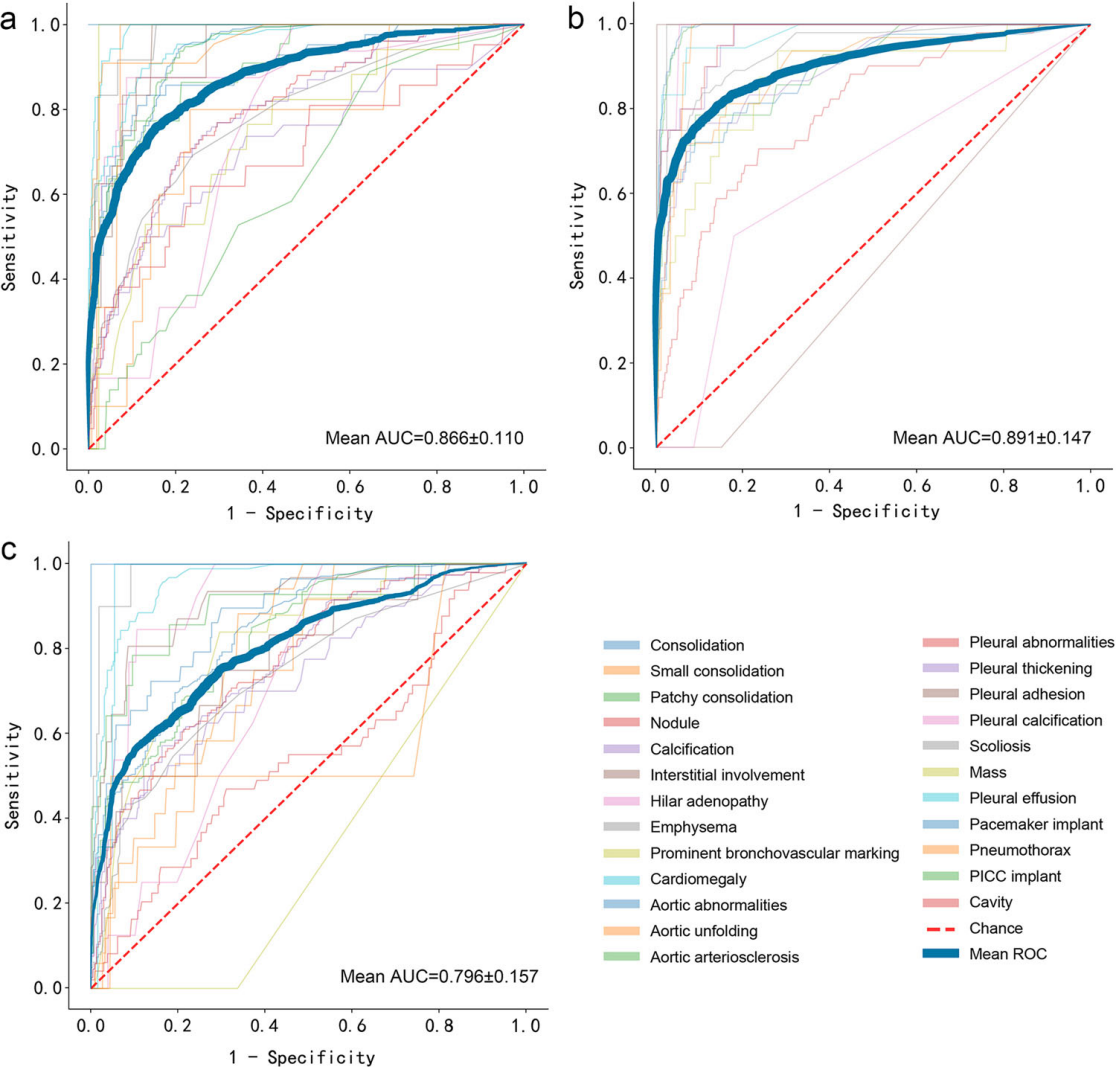
Receiver operating characteristic (ROC) curves of 25 abnormal signs on CXR in the three external test cohorts. **a** In the cohort of symptomatic patients in the academic hospital, the mean AUC was 0.866 ± 0.110. The AUCs of major abnormal signs, i.e., consolidation, nodule, mass, pneumothorax, and pleural effusion, were 0.900 (95% CI: 0.849–0.943), 0.698 (0.581–0.806), 0.977 (0.965–0.988), 0.963 (0.925–0.991), and 0.988 (0.980–0.994), respectively. **b** In the cohort of asymptomatic screening examinees in the academic hospital, the mean AUC was 0.891 ± 0.147. The AUCs of common signs, i.e., consolidation and nodule, were 0.876 (0.817–0.920) and 0.796 (0.725–0.838), respectively. **c** In the cohort of symptomatic patients in eight community clinics, the mean AUC was 0.796 ± 0.157. The AUCs of major signs, i.e., consolidation, nodule, and mass, were 0.873 (95% CI: 0.815–0.926), 0.698 (0.619–0.771), and 1.000 (0.991–1.000), respectively.

**Fig. 4:**
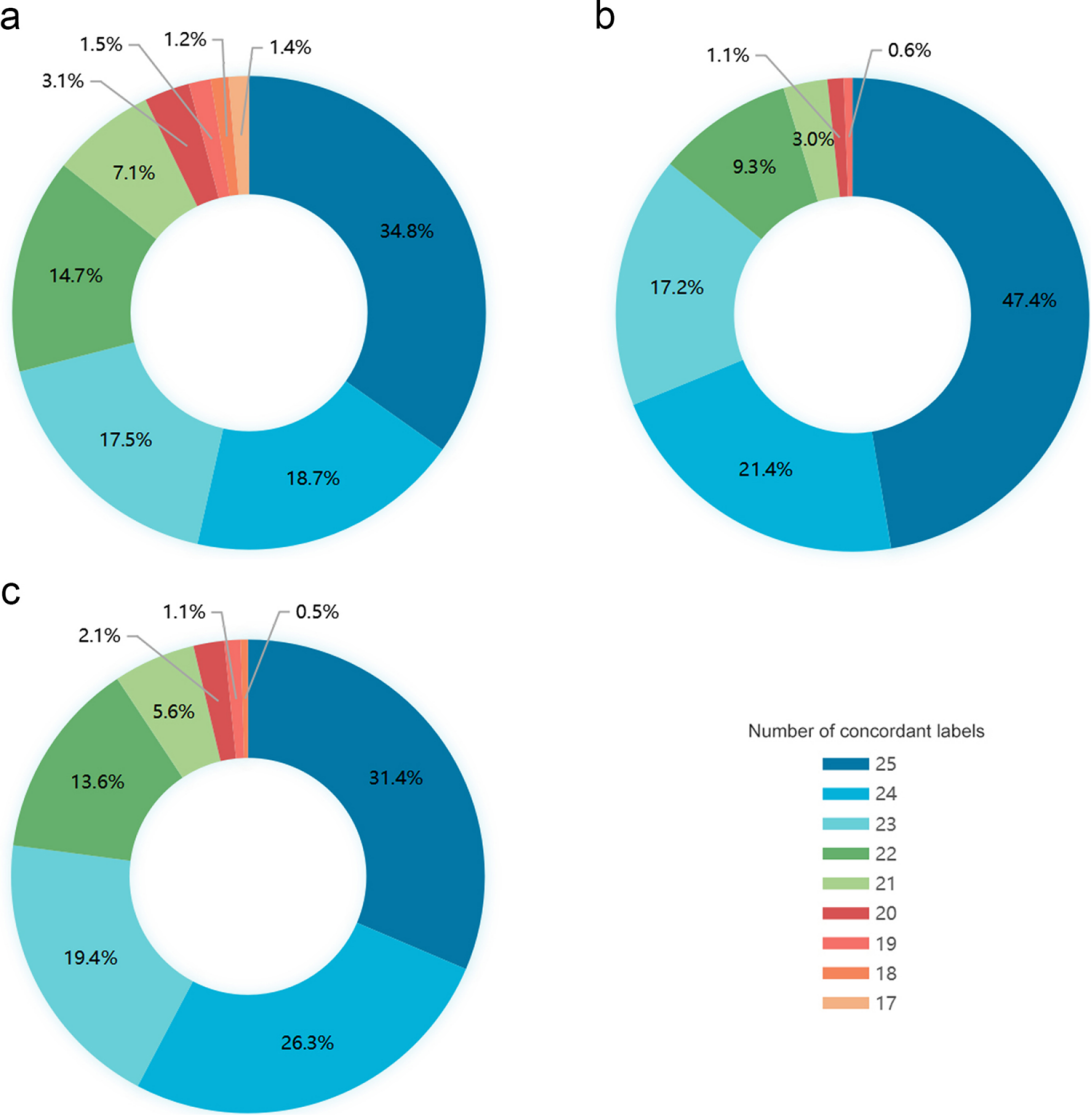
Number and percentage of concordant labels between the convolutional neural network (CNN) and expert consensus reading in the three test cohorts. **a** In the cohort of symptomatic patients in the academic hospital, 2092 patients (34.8%) showed consistency on 25 signs between CNN and the consensus reading, and 1121 (18.7%), 1049 (17.5%), and 881 (14.7%) patients showed consistency on 24, 23, and 22 signs, respectively. Overall, CNN correctly classified ≥22 (88%) abnormal signs in 5142 patients (85.8%). **b** In the cohort of asymptomatic screening examinees in the academic hospital, 1010 (47.4%), 456 (21.4%), 366 (17.2%), and 198 (9.3%) patients showed consistency on 25, 24, 23, and 22 signs between CNN and the consensus reading, respectively. Overall, CNN correctly classified ≥22 signs in 2030 (95.3%) patients. **c** In the cohort of symptomatic patients in eight community clinics, 566 (31.4%), 474 (26.3%), 350 (19.4%), and 245 (13.6%) patients showed consistency between CNN and the consensus reading on 25, 24, 23, and 22 labels, respectively. Overall, CNN correctly classified ≥22 (88%) abnormal signs in 1636 (90.7%) patients.

**Fig. 5:**
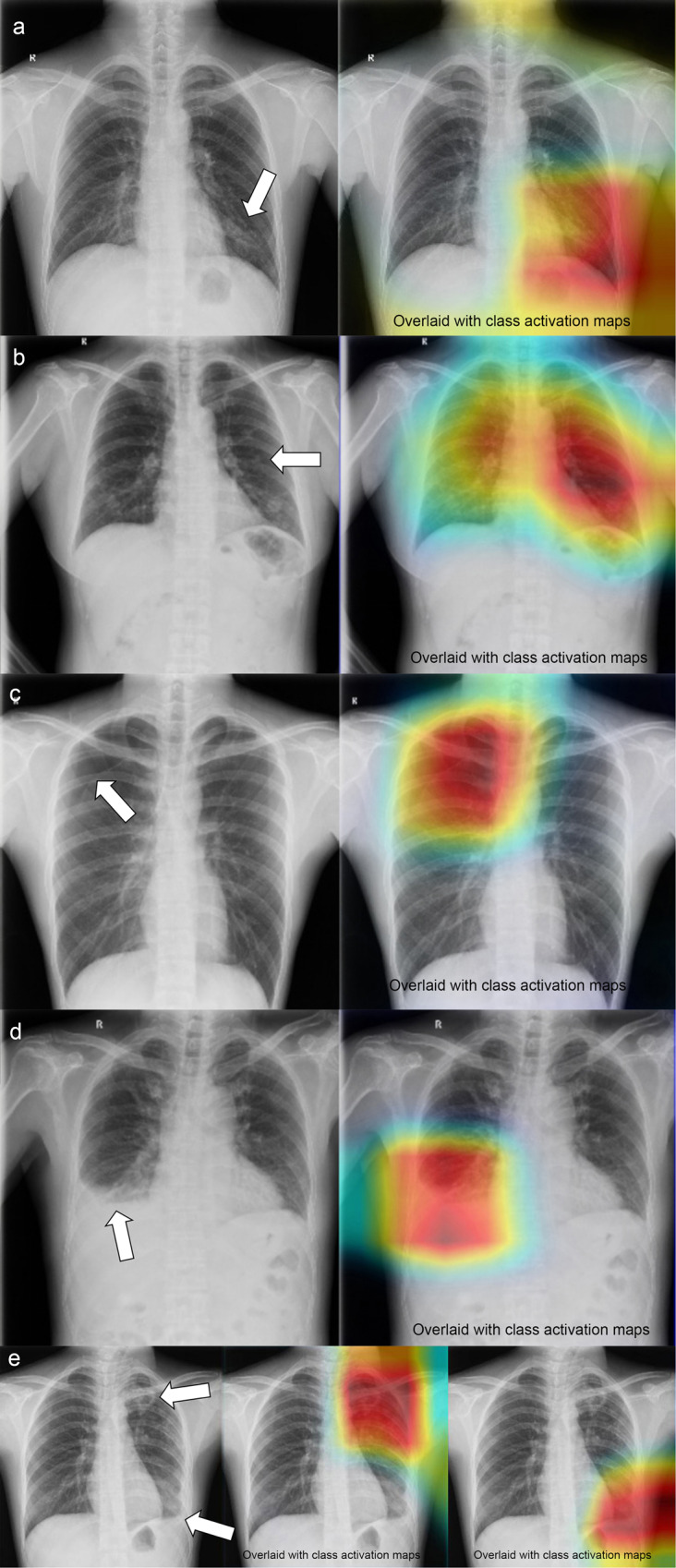
Representative chest radiographs overlaid with class activation maps (CAM) showing the active area of convolutional neural network (CNN). **a** The inferior lung field of the left lung is overlaid by CAM, which reveals a patchy density (white arrow). The CNN noted that this case had a “patchy consolidation” sign, while the other 24 abnormal signs were absent. **b** The middle and lower fields of the left lung are overlaid by CAM, which reveals a pulmonary nodule (white arrow). The CNN indicated that this case had a “nodule” sign, while the other 24 abnormal signs were absent. **c** The upper right lung is overlaid by CAM. The CNN indicated that this case has a “pneumothorax” label. The radiologists confirmed this finding and found visible visceral pleural margins (white arrow), but there was no lung texture outside this line. The other 24 abnormal signs were absent. **d** The lower right lung is overlaid by CAM. The CNN indicated that this case has a “hydrothorax” sign. The radiologists confirmed this finding and identified an air-fluid level (white arrow). **e** The upper field and lower field of the left lung are overlaid by CAMs. The CNN indicated that this case had “patchy consolidation” and “pleural effusion” signs. The other 23 abnormal signs were absent. The radiologists confirmed CNN’s findings (white arrows).

The consensus reading of experienced radiologists was used as a reference for testing CNN’s classification performance. Fig. 3 (Supplementary Data 3) shows the receiver operating characteristic (ROC) curves for the three test cohorts. Because the number of correctly classified labels of CNN determines its ability to interpret CXR, we further evaluated the number of consistent labels between CNN and consensus reading (Fig. 4 and Supplementary Data 4). Figure 5 displays several example CXR cases with AI classification.

In the symptomatic patients (*n* = 5996) from Hospital-2, all 25 abnormal signs were present. The mean AUC, accuracy, sensitivity, specificity, and F1-score of CNN reached 0.866 ± 0.110, 0.907 ± 0.081, 0.631 ± 0.245, 0.919 ± 0.079, and 0.716 ± 0.189, respectively (Supplementary Data 5). The AUCs of major abnormal signs, i.e., consolidation, nodule, mass, pneumothorax, and pleural effusion, were 0.900 (95% CI: 0.849–0.943), 0.698 (0.581–0.806), 0.977 (0.965–0.988), 0.963 (0.925–0.991), and 0.988 (0.980–0.994), respectively. The accuracies of these signs were 0.935 (0.908–0.955), 0.930 (0.903–0.951), 0.974 (0.955–0.986), 0.970 (0.949–0.982), and 0.970 (0.949–0.982), respectively.

In this cohort, 2092 patients (34.8%) showed consistency on 25 signs between CNN and the consensus reading, and 1121 (18.7%), 1049 (17.5%), and 881 (14.7%) patients showed consistency on 24, 23, and 22 signs, respectively. Overall, CNN correctly classified ≥22 (88%) abnormal signs in 5142 patients (85.8%).

The AUC was not significantly different in identifying 21 abnormal signs between CNN and the local radiologists in an academic hospital (Delong’s *p* > 0.05). But the AUC of CNN was lower than that of radiologists in determining small consolidation (*p* = 0.002), nodule (*p* < 0.001), calcification (*p* < 0.001), and PICC implant (*p* < 0.001).

### Screening examinees from an academic hospital

In the asymptomatic screening examinees (*n* = 2130) from Hospital-2, 18 abnormal signs were present, while the other seven were absent, including mass, cavity, edema, pneumothorax, pleural effusion, PICC implant, and pacemaker implant. The mean AUC, accuracy, sensitivity, specificity, and F1-score of CNN were 0.891 ± 0.147, 0.925 ± 0.040, 0.523 ± 0.259, 0.984 ± 0.006, and 0.640 ± 0.248, respectively (Supplementary Data 6). The AUCs of common signs, i.e., consolidation and nodule, were 0.876 (0.817–0.920) and 0.796 (0.725–0.838), respectively. The accuracies were 0.976 (0.969–0.982) and 0.977 (0.970–0.983), respectively.

In this cohort, 1010 (47.4%), 456 (21.4%), 366 (17.2%), and 198 (9.3%) patients showed consistency on 25, 24, 23, and 22 signs between CNN and the consensus reading (the absent signs were compared according to the negative results), respectively. Overall, CNN correctly classified ≥22 signs in 2030 (95.3%) patients.

The AUC was not significantly different in determining 17 abnormal signs between CNN and local radiologists (all *p* > 0.05). But the AUC of CNN was lower than that of radiologists in determining nodule (*p* = 0.013).

### Patients from eight community clinics

In the symptomatic patients (*n* = 1804) from eight community clinics, 21 abnormal signs were present. The other four were absent, including cavity, edema, pneumothorax, and PICC implant. The mean AUC, accuracy, sensitivity, specificity, and F1-score of CNN were 0.796 ± 0.157, 0.861 ± 0.106, 0.609 ± 0.297, 0.866 ± 0.189, and 0.674 ± 0.228, respectively (Supplementary Data 7). The AUCs of major signs, i.e., consolidation, nodule, and mass, were 0.873 (95% CI: 0.815–0.926), 0.698 (0.619–0.771), and 1.000 (0.991–1.000), respectively. The accuracies were 0.916 (0.881–0.941), 0.672 (0.624–0.720), and 1.000 (0.991–1.000), respectively.

In this cohort, 566 (31.4%), 474 (26.3%), 350 (19.4%), and 245 (13.6%) patients showed consistency between CNN and the consensus reading on 25, 24, 23, and 22 labels, respectively. Overall, CNN correctly classified ≥22 (88%) abnormal signs in 1636 (90.7%) patients. The absent signs were compared according to the negative results.

The AUC was not significantly different in identifying 12 abnormal signs between CNN and the local radiologists in community clinics (all *p* > 0.05). The AUC of CNN was higher than that of local radiologists in identifying hilar adenopathy, thickened bronchovascular markings, cardiomegaly, abnormal aorta, aortic unfolding, and abnormal pleura (all *p* < 0.001), but lower in identifying calcification (*p* < 0.001).

## Discussion

In this study, we used the BERT model to extract linguistic entities and relationships from unstructured radiology reports, and established 25 labels representing 25 abnormal signs found on CXR, so as to train CNN with weakly supervised labeling. In the three external test cohorts of symptomatic patients, screening examinees, and community clinic patients, the mean AUC of CNN for the 25 labels reached 0.866, 0.891, and 0.796, respectively. In particular, the mean specificity was as high as 0.919, 0.974, and 0.866, indicating a very low false-positive rate that is potentially helpful to screen out patients with abnormal signs on CXR. The classification performance of CNN was slightly lower than that of local radiologists in an academic hospital but slightly higher than that in community clinics.

Multiple AI studies aimed at disease detection and classification on CXR. Classifying lesions on CXR has been widely explored, the size of publicly released datasets is increasing^33, 34^. Recently, two large datasets have been made publicly available. The Open-I dataset contains 7470 CXR cases with 3955 radiology reports, but the images were not explicitly annotated by disease category^35^. The ChestX-ray14 dataset consists of 112,120 frontal-view CXR images from 30,805 patients^36^. The developer of the ChestX-ray14 dataset proposed a 14-label classification system, using traditional NLP technology to extract keywords from radiology reports to classify chest diseases^14, 36^. Thereafter, NLP technology has shown the advantage of automatically labeling a large number of CXR images, greatly reducing the difficulty and workload of image annotation. The BERT model has made remarkable achievements in processing common language, such as for the credibility analysis of information on social media^37, 38^. In this study, this model proved its ability to process medical terms in radiology reports, thus laying the foundation for establishing a 25-label classification system on CXR.

After extracting linguistic entities and relationships from unstructured radiology reports, we established 25 labels of abnormal signs, which reflected the distribution of common abnormal signs in 74,082 patients. Of the 25 labels, seven were frequently used in previous studies, i.e., consolidation, nodule, mass, edema, effusion, emphysema, and pneumothorax; the other 18 were not commonly used as labels in AI research but were frequently used in diagnostic reports, such as hilar adenopathy, thickened bronchovascular markings, interstitial disease, abnormal aorta, abnormal pleura, scoliosis, and pacemaker implant. Although other studies have applied more labels^13, 15^, this comprehensive labeling system can reflect the abnormal signs in actual diagnostic reports.

We conducted a comprehensive external test on two levels of medical institutes (academic hospital vs. community clinics) and two types of patients (symptomatic patients vs. screening examinees). Most of the datasets of previous AI studies on CXR came from academic hospitals, such as the ChestX-ray14 dataset released by the National Institutes of Health Clinical Center^39^. A recent study on the classification of abnormal CXR used the Rhode Island Hospital chest radiograph (RIH-CXR) database^40^, from an academic hospital, but the cases in this database were only marked as normal or abnormal. Due to the different distribution of chest diseases in academic hospitals and community clinics, it is reasonable to separate the investigation of academic hospitals and community clinics. Our results have suggested that CNN performed well in detecting abnormal signs on CXR in these two levels of medical institutions. In addition, we conducted a test on the screening population. The positive rate of abnormal signs on CXR in asymptomatic screening examinees was significantly lower than that of symptomatic patients (9.7% vs. 44.8%), and the types of diseases were less. In this screening scenario, the average AUC and accuracy of CNN were 0.891 and 0.925, respectively. As far as we know, no other study has verified the performance of CNN for identifying multiple disorders on CXR in symptomatic patients and asymptomatic screening examinees. Our methods and results are more in line with the need of applying AI approach to different types of patients in practice.

Interestingly, CNN’s performance was only slightly lower than that of local radiologists in an academic hospital, but marginally higher than that in community clinics. This difference may be that the radiologists in community clinics usually have less experience than those in academic hospitals^2^. In addition, we found that CNN had more than 22 correct classifications out of 25 for 86% of patients in academic hospitals and 91% of patients in community clinics. These results indicate that CNN can identify most of the common diseases found on CXR, which provides a prospect for clinical application.

The World Health Organization emphasized the potential clinical value of CXR in screening and diagnosing pulmonary and cardiac diseases, and pointed out the lack of diagnostic expertise in primary hospitals^41^. Even experienced radiologists have limitations in their ability, such as cognitive and perceptual biases and fatigue, all of which can lead to diagnostic errors^42^. CNN approach can at least be used as an auxiliary tool for radiologists, which could help to reduce misinterpretation. Khosravan et al. showed that a collaborative computer-aided diagnosis system can help radiologists reduce diagnostic errors^43^. Kundel et al. showed that perceptual biases and diagnostic errors can be decreased by giving feedback about abnormal signs on CXR to radiologists^44^. This clinical strategy fits well with our proposed CNN approach. AI assistant system can screen abnormal signs on CXR, as a prereading tool for radiologists to evaluate CXR, to improve reading efficiency and accuracy. Our research allows the diagnosis of 25 abnormal signs on CXR, which is essential in large academic hospitals with heavy workloads, and community clinics with relatively inexperienced medical staff.

Although CT screening is recommended and widely used in the present medical environments, CXR retains an important screening role in many countries. Furthermore, in some cases, lung cancer was detected by chest CT but was retrospectively identified as a missed finding on prior CXR^45^. The population without CT examination mainly relies on CXR to screen and diagnose chest and cardiac diseases. The AI-based system can improve the workflow, especially for screening units with many subjects but a low true-positive rate.

This study has some limitations. First, we only trained CNN on posteroanterior-view CXR. A previous study reported that lateral-view CXR improved the diagnostic accuracy in 15% of cases^1^. Second, in completing the radiology reports, radiologists observe CXR images, patient characteristics, and medical history, but CNN only views the images based on the image labels extracted from radiology reports. Additional analysis of patient characteristics and medical history will improve AI’s ability to interpret CXR cases, especially for some special diseases^46^. Third, there may be errors in radiology reports and label extraction. Although we have iteratively corrected them through knowledge graphs and expert panel discussions, it is necessary to conduct a prospective study to evaluate the influence of errors on reporting. Fourth, the positive rate of some abnormal signs was low, so the imbalance of training data may affect the performance of CNN. Some rare diseases or infrequent abnormal signs, such as edema and cavity, may not be fully learned by CNN. Providing a balanced dataset for each disease label will improve the robustness and generalizability of CNN^47^. Finally, the current study was conducted in one country. The performance of CNN needs to be further validated in other populations.

In this study, we developed and validated an AI approach to interpret CXR based on the BERT NLP model to extract linguistic entities and relationships from radiology reports to establish a 25-label system of abnormal signs on CXR. In a multicenter external test setting of different populations, the CNN method achieved high AUC and specificity in identifying abnormal signs on CXR, and its classification performance was slightly lower than that of local radiologists in an academic hospital but marginally higher than those in community clinics. The CNN-derived classification of most abnormal signs was consistent with expert consensus reading. Thus, AI-assisted interpretation of CXR images may enhance the diagnostic capability and efficiency of doctors for common disorders, especially for primary hospitals short of radiologists or academic medical centers with heavy workloads. This study suggests the possibility of using NLP to construct an effective CXR report annotation system, which improves the annotation efficiency of large-sample images and then inspires the use of NLP in other medical image annotations.

### Reporting Summary

Further information on research design is available in the Nature Research Reporting Summary linked to this article.

## Supplementary information

Supplementary Information

Supplementary Data 1

Supplementary Data 2

Supplementary Data 3

Supplementary Data 4

Supplementary Data 5

Supplementary Data 6

Supplementary Data 7

Description of Additional Supplementary Files

Reporting Summary

## Data Availability

Source data for the main figures in the manuscript can be accessed as Supplementary Data 1, 3, and 4. The test data of CRADI datasets (raw data) are available only for scientific purposes at https://zenodo.org/record/5493595#.YTmMPBniuUk/^20^ The training dataset is not publicly available to protect patient privacy, since this dataset contains patient-identifiable information. If any researcher wants to use the training dataset for scientific purposes, please contact the corresponding author and apply for ethical approval from the data provider.

## Code availability

Codes and demo data for this study are available at https://zenodo.org/record/5335914^30^. The code and model of the bidirectional encoder representations from transformers (BERT) are at https://github.com/google-research/bert/. We performed this study with open-source tools, including a deep learning library (PyTorch 0.4.0, http://pytorch.org/), a computer vision library (opencv, http://opencv.org/), and a data analysis library (scikit-learn, http://scikit-learn.org/).
