# Supplementary material for: Development and multicenter validation of chest X-ray radiography interpretations based on natural language processing
Source: Commun Med (Lond). 2021 Oct 28;1:43. doi: 10.1038/s43856-021-00043-x (PMC9053275; doi:10.1038/s43856-021-00043-x)
Supplement: Supplementary file 1 — Supplementary Information [file 43856_2021_43_MOESM1_ESM.pdf]

Supplementary Figure 1 Representative linguistic entities and relationships extracted by the bidirectional encoder representations from transformers (BERT) model from unstructured radiology reports

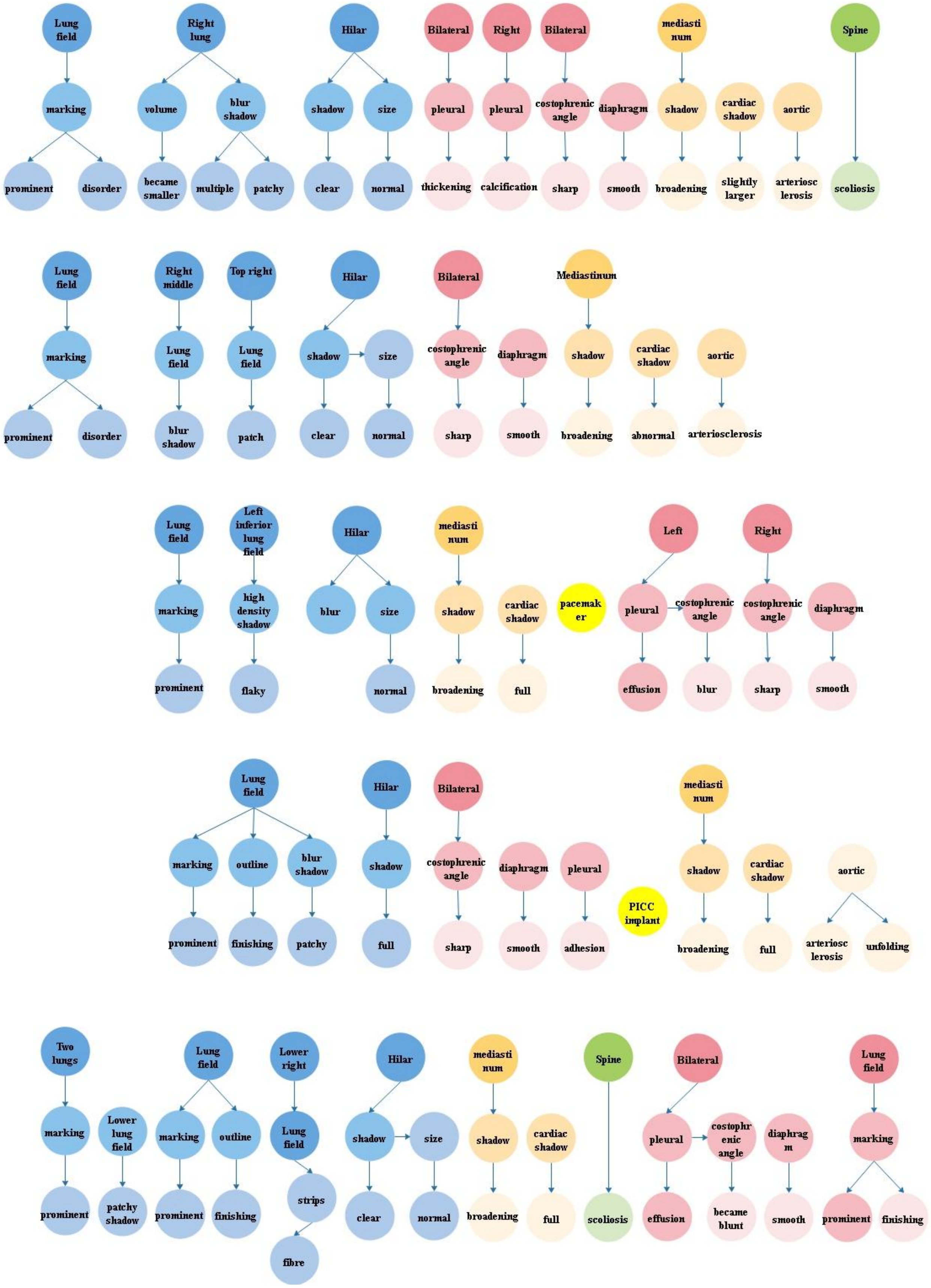

Supplementary Figure 2 Visualization of label distribution in the training and test datasets by t-distributed stochastic neighbor embedding (t-SNE). According to the joint probability principle, the weights of the main positive labels were summed to calculate the color index. Each point represents a subject. The larger number in this color spectrum, the more abnormal labels the subject has. In this way, magenta color represents more abnormal signs, and deep blue color represents no or less abnormal signs. A) symptomatic patients in Hospital-1 (*n*=74,082); B) symptomatic patients from Hospital-2 (*n*=5,996); C) asymptomatic screening examinees from Hospital-2 (*n*=2,130); D) symptomatic patients from eight community clinics (*n*=1,804).

AN = all negative; PL = pleura; LP = lung parenchyma; ME = mediastinum; TH = thoracic wall

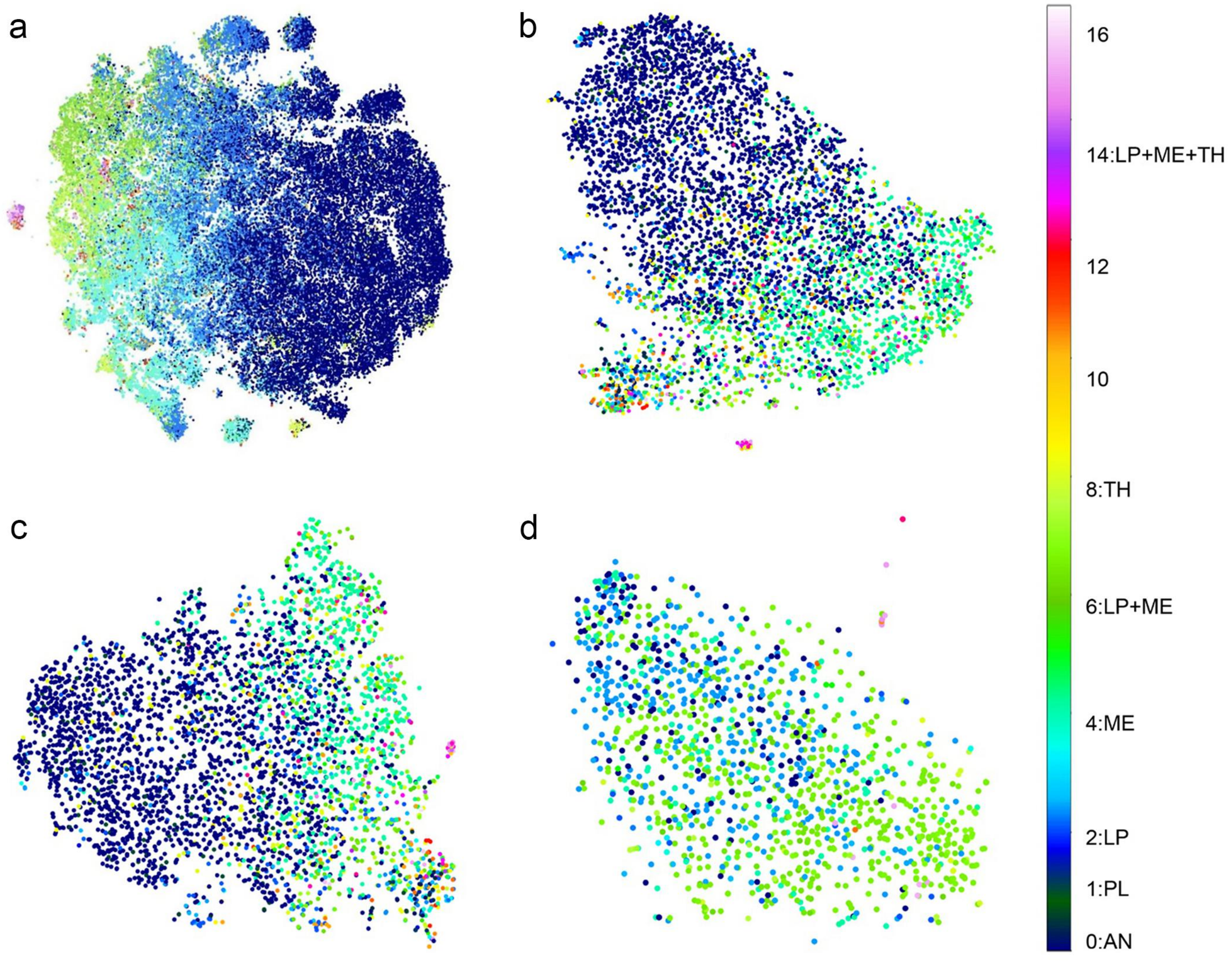

Supplementary Table 1 Digital X-ray radiography systems used in the Chest Radiography at Diverse Institutes (CRADI) dataset

Institution		Patients, *n*	Model of digital radiography	Tube voltage, kVp	Image matrix
Hospital-1 (*n*=74,802)		66,054	Philips DigitalDiagnost	74	2048×2048
		386	Samsung GC85A	Unknown	2048×2048
		362	Shimadzu RADspeed	125	2048×2048
		11,187	Canon CXDI	125	2048×2048
		35	Optima XR220	Unknown	2048×2048
Hospital-2 (*n*=8,126)	Patients	5,950	GE Discovery XR656	80-120	2048×2048
		22	GE Optima XR220	55	2048×2048
		24	DMS Platinum 43	80	2048×2048
	Screening	1,007	GE Discovery XR656	120	2048×2048
		1,123	Canon CXDI	120	2048×2048
Eight community clinics (*n*=1,804)		553	Canon CXDI	77	2048×2048
		841	Carestream DRX-Evolution	80-110	2048×2048
		110	United Imaging uDR	80	2048×2048
		112	Siemens Fluorospot Compact FD	84.8	2048×2048
		188	Philips Digital Diagnost	125	2048×2048
