# Supplementary material for: Development and multicenter validation of chest X-ray radiography interpretations based on natural language processing
Source: Commun Med (Lond). 2021 Oct 28;1:43. doi: 10.1038/s43856-021-00043-x (PMC9053275; doi:10.1038/s43856-021-00043-x)
Supplement: Supplementary file 9 — Description of Additional Supplementary Files [file 43856_2021_43_MOESM9_ESM.pdf]

Description of Additional Supplementary Files

File Name: Supplementary Data 1

Description: Label distribution data of the training and three test cohorts (patients and screening examinees in Hospital-2, and patients from eight community clinics) by t-distributed stochastic neighbor embedding (t-SNE). t-SNE algorithm reduced the dimension of abnormal signs of four anatomical locations into a two-dimensional plane. In the datasheet, the x and y coordinates represent the position of the point in the two-dimensional plane, and the value indicates the weighted color.

File Name: Supplementary Data 2

Description: Classification performance of convolutional neural network (CNN) in the training cohort from Hospital-1 (n=74,082).

File Name: Supplementary Data 3

Description: Receiver operating characteristic (ROC) curve data of the three test cohorts (patients and screening examinees in Hospital-2, and patients from eight community clinics).

File Name: Supplementary Data 4

Description: Number and percentage of concordant labels between the convolutional neural nertwork (CNN) and expert consensus reading in the three test cohorts (patients and screening examinees in Hospital-2, and patients from eight community clinics).

File Name: Supplementary Data 5

Description: Classification performance between the convolutional neural network (CNN) and local radiologists in the symptomatic patients from Hospital-2 (n=5,996).

File Name: Supplementary Data 6

Description: Classification performance between the convolutional neural network (CNN) and local radiologists in asymptomatic screening examinees from Hospital-2 (n=2,130).

File Name: Supplementary Data 7

Description: Classification accuracy between the convolutional neural network (CNN) and local radiologists in symptomatic patients from eight community clinics (n=1,804).
