# Supplementary material for: Development and multicenter validation of chest X-ray radiography interpretations based on natural language processing
Source: Commun Med (Lond). 2021 Oct 28;1:43. doi: 10.1038/s43856-021-00043-x (PMC9053275; doi:10.1038/s43856-021-00043-x)
Supplement: Supplementary file 10 — Reporting Summary [file 43856_2021_43_MOESM10_ESM.pdf]

nature research

Corresponding author(s): Xueqian Xie
Last updated by author(s): Sep 9, 2021

Reporting Summary

Nature Research wishes to improve the reproducibility of the work that we publish. This form provides structure for consistency and transparency in reporting. For further information on Nature Research policies, see our Editorial Policies and the Editorial Policy Checklist.

Statistics

For all statistical analyses, confirm that the following items are present in the figure legend, table legend, main text, or Methods section.

n/a	Confirmed	
☐	☒	The exact sample size (*n*) for each experimental group/condition, given as a discrete number and unit of measurement
☐	☒	A statement on whether measurements were taken from distinct samples or whether the same sample was measured repeatedly
☐	☒	The statistical test(s) used AND whether they are one- or two-sided<br>*Only common tests should be described solely by name; describe more complex techniques in the Methods section.*
☒	☐	A description of all covariates tested
☐	☒	A description of any assumptions or corrections, such as tests of normality and adjustment for multiple comparisons
☐	☒	A full description of the statistical parameters including central tendency (e.g. means) or other basic estimates (e.g. regression coefficient) AND variation (e.g. standard deviation) or associated estimates of uncertainty (e.g. confidence intervals)
☐	☒	For null hypothesis testing, the test statistic (e.g. *F*, *t*, *r*) with confidence intervals, effect sizes, degrees of freedom and *P* value noted<br>*Give P values as exact values whenever suitable.*
☒	☐	For Bayesian analysis, information on the choice of priors and Markov chain Monte Carlo settings
☒	☐	For hierarchical and complex designs, identification of the appropriate level for tests and full reporting of outcomes
☒	☐	Estimates of effect sizes (e.g. Cohen's *d*, Pearson's *r*), indicating how they were calculated

Our web collection on statistics for biologists contains articles on many of the points above.

Software and code

Policy information about availability of computer code

Data collection	NA
Data analysis	Codes and demo data for this study are available at https://doi.org/10.5281/zenodo.5335914/. The code and model of the bidirectional encoder representations from transformers (BERT) are at https://github.com/google-research/bert. We performed this study with open-source tools, including a deep learning library (PyTorch 0.4.0, http://pytorch.org/), a computer vision library (opencv, http://opencv.org/) and a data analysis library (scikit-learn, http://scikit-learn.org/).

For manuscripts utilizing custom algorithms or software that are central to the research but not yet described in published literature, software must be made available to editors and reviewers. We strongly encourage code deposition in a community repository (e.g. GitHub). See the Nature Research guidelines for submitting code & software for further information.

Data

Policy information about availability of data

All manuscripts must include a data availability statement. This statement should provide the following information, where applicable:

- Accession codes, unique identifiers, or web links for publicly available datasets
- A list of figures that have associated raw data
- A description of any restrictions on data availability

Source data for the main figures in the manuscript can be accessed as Supplementary Data 1, 3, and 4. The Chest Radiography at Diverse Institutes (CRADI) datasets (raw data) of this study are available at https://zenodo.org/record/5493595#.YTmMPBniuUk/.

Field-specific reporting

Please select the one below that is the best fit for your research. If you are not sure, read the appropriate sections before making your selection.

☒ Life sciences ☐ Behavioural & social sciences ☐ Ecological, evolutionary & environmental sciences

For a reference copy of the document with all sections, see nature.com/documents/nr-reporting-summary-flat.pdf

Life sciences study design

All studies must disclose on these points even when the disclosure is negative.

Sample size	The population as the training dataset (n=74,082) was retrospectively and consecutively collected from February 2014 to February 2018 in an academic hospital (Hospital-1). The cases in CRADI dataset as external test data came from another academic hospital (Hospital-2) (n=7,797) and eight community clinics (n=1,804).
Data exclusions	The exclusion criteria were: mobile CXR, insufficient image quality, and incomplete reports without the confirmation of a senior radiologist.
Replication	Two radiologists with 21 and 31 years of experience independently accessed the CXR images and BERT-extracted labels. They made necessary corrections to the label according to their clinical experience and resolved the inconsistency by consensus.
Randomization	NA
Blinding	NA

Reporting for specific materials, systems and methods

We require information from authors about some types of materials, experimental systems and methods used in many studies. Here, indicate whether each material, system or method listed is relevant to your study. If you are not sure if a list item applies to your research, read the appropriate section before selecting a response.

Materials & experimental systems

n/a	Involved in the study	
☒	☐	Antibodies
☒	☐	Eukaryotic cell lines
☒	☐	Palaeontology and archaeology
☒	☐	Animals and other organisms
☐	☒	Human research participants
☒	☐	Clinical data
☒	☐	Dual use research of concern

Methods

n/a	Involved in the study	
☒	☐	ChIP-seq
☒	☐	Flow cytometry
☒	☐	MRI-based neuroimaging

Human research participants

Policy information about studies involving human research participants

Population characteristics	Adults ≥18 years old with symptoms such as fever, cough, and chest pain, as indications for CXR.<br>The subjects of screening participants were asymptomatic.
Recruitment	The CXR image and corresponding diagnostic report of each subject were retrieved from the picture archiving and communication system (PACS) and radiology information system (RIS).
Ethics oversight	Local IRB of Shanghai General Hospital

Note that full information on the approval of the study protocol must also be provided in the manuscript.
